# Mind the gap—deficits in our knowledge of aspects impacting the bioavailability of phytochemicals and their metabolites—a position paper focusing on carotenoids and polyphenols

**DOI:** 10.1002/mnfr.201400745

**Published:** 2015-06-03

**Authors:** Torsten Bohn, Gordon J. McDougall, Amparo Alegría, Marie Alminger, Eva Arrigoni, Anna‐Marja Aura, Catarina Brito, Antonio Cilla, Sedef N. El, Sibel Karakaya, Marie C. Martínez‐Cuesta, Claudia N. Santos

**Affiliations:** ^1^ Environmental Research and Innovation Department, Luxembourg Institute of Science and TechnologyBelvauxLuxembourg; ^2^ The James Hutton InstituteInvergowrieDundeeUnited Kingdom; ^3^ Nutrition and Food Science AreaFaculty of Pharmacy, University of ValenciaAv. Vicente Andrés Estellés s/nBurjassotValenciaSpain; ^4^ Department of Chemical and Biological EngineeringChalmers University of TechnologyGothenburgSweden; ^5^ Agroscope, Institute for Food Sciences (IFS)WädenswilSwitzerland; ^6^ VTT Technical Research Centre of FinlandEspooFinland; ^7^ IBET, Instituto de Biologia Experimental e TecnológicaOeirasPortugal; ^8^ Instituto de Tecnologia Química e Biológica António XavierUniversidade Nova de LisboaOeirasPortugal; ^9^ Ege UniversityEngineering Faculty, Food Engineering DepartmentIzmirTurkey; ^10^ Instituto de Investigación en Ciencias de la Alimentación CIAL (CSIC‐UAM)MadridSpain

**Keywords:** Biotransformation, Food processing, Microbiota, Mixed diet, Transporters

## Abstract

Various secondary plant metabolites or phytochemicals, including polyphenols and carotenoids, have been associated with a variety of health benefits, such as reduced incidence of type 2 diabetes, cardiovascular diseases, and several types of cancer, most likely due to their involvement in ameliorating inflammation and oxidative stress. However, discrepancies exist between their putative effects when comparing observational and intervention studies, especially when using pure compounds. These discrepancies may in part be explained by differences in intake levels and their bioavailability. Prior to exerting their bioactivity, these compounds must be made bioavailable, and considerable differences may arise due to their matrix release, changes during digestion, uptake, metabolism, and biodistribution, even before considering dose‐ and host‐related factors. Though many insights have been gained on factors affecting secondary plant metabolite bioavailability, many gaps still exist in our knowledge. In this position paper, we highlight several major gaps in our understanding of phytochemical bioavailability, including effects of food processing, changes during digestion, involvement of cellular transporters in influx/efflux through the gastrointestinal epithelium, changes during colonic fermentation, and their phase I and phase II metabolism following absorption.

**Abbreviations**
CVD: cardiovascular diseases
MCTs: monocarboxylic acid transporters
MRP: multidrug resistance protein
NEPP: nonextractable polyphenols
NPC1L1: Niemann‐Pick C1‐Like 1
SGLT‐1: sodium‐glucose linked transporter 1
T2D: type 2 diabetes
TOF: time‐of‐flight

## 1 Introduction

Phytochemicals comprise a diverse group of secondary plant compounds, including polyphenols, carotenoids, triterpenes, phytosterols, glucosinolates, and many more. These compounds have aroused increasing interest in the area of nutrition and food science, due to their potential health benefits. Several epidemiological studies have suggested that their consumption or tissue concentration is associated with reduced risk of developing certain chronic diseases, namely, type 2 diabetes (T2D), cardiovascular diseases (CVD), and some types of cancer. For example, meta‐analyses have suggested that the consumption of flavonoids from fruits and vegetables improved flow‐mediated dilation, a marker of atherosclerosis 1, and suggested positive health effects of consuming carotenoids with respect to type 2 diabetes 2.

However, though a few examples of intervention studies with isolated compounds, e.g. resveratrol, or curcumin 3, 4, producing positive health effects exist, micronutrients and phytochemicals may not act efficiently in isolation but together with many other compounds in the food matrix, leading to synergistic effects 5. It is thus important to determine efficacy, safety and underlying mechanisms of these compounds, especially when taken in pharmaceutical doses/in combination with other drugs. For example, Prasain et al. 6 reviewed risks and benefits of dietary versus supplemented/isolated flavonoids, stating that flavonoids can be detrimental in some settings and therefore are not universally safe. Adverse effects were also found following the ingestion of beta‐carotene supplements administrated to CVD patients 7, 8, and vitamin C and E as well as beta‐carotene supplementation failed to show health benefits with respect to CVD 9. Supplementing individual flavonoids and isoflavonoids has also been met with criticism, e.g. due to endocrine disrupting properties for higher doses 10. The reasons for this discrepancy are not fully understood, but could involve “missing” synergistic effects with the food matrix, altered digestion and release of the compounds or changed degradation patterns, i.e. modified bioavailability (the fraction of a compound that is absorbed and can be used for physiological functions and/or storage). Indeed, many reviews on bioavailability and bioactivity of phytochemicals are available 11, 12, 13. Phytochemicals may have multiple functions in the human body, and depending on their dose they can exert both beneficial and deleterious effects 14.

This position paper attempts to identify the aspects of digestion, release, absorption and metabolism of food phytochemicals which are only poorly understood (Table 1) 15. Even prior to ingestion processing factors (food texture, e.g. heat, temperature, or pressure application 16) can impinge on the bioaccessibility (fraction of a compound that is released from the matrix and potentially available for further uptake and absorption) of bioactives 17. Similarly, the influence of consuming a mixed diet, i.e. “real” complex meals, on bioaccessibility and absorption is poorly comprehended, and biochemical and physico‐chemical aspects such as viscosity and surface tension surely play a role 18. Following ingestion, enzyme concentrations, pH, and time of digestion all play a role and influence release kinetics and degradation patterns 19, 20. A factor that is also poorly understood is the nature and bioactivity of metabolites formed during digestion, as following their fate is challenging. However, the bioavailability of some compounds and their metabolites may be higher than previously assumed, as shown, e.g. in isotope studies with anthocyanins 21. Some lipophilic compounds, such as carotenoids or triterpenes, require micellarization, as may certain polyphenol aglycones 22. Following release, pathways of absorption, i.e. active versus passive or paracellular routes remain largely marginally understood. The same is true for influx and efflux transporters in the epithelium. For example, several polyphenols were shown to be considerably better absorbed in the presence of additional polyphenols, blocking efflux transporters (to the gut), which normally reduce the intracellular concentration of such “xenobiotics” 19. On the other hand, transporters to the basolateral side are hardly understood. Finally, many native phytochemicals undergo considerable metabolism in the human body, e.g. deglycosylation and glucuronidation/sulfation for polyphenols in the gut, cleavage by beta‐carotene oxygenase 1 and beta‐carotene dioxygenase 2 for carotenoids 23, and many additional reactions may occur in other tissues such as in the liver or in the colon, where bacterial fermentation significantly alters the structure and profile and thus the potential bioactivity of many plant compounds that are not absorbed in the small intestine 24, 25, 26.

**Table 1:** Summary of major gaps of knowledge around phytochemical bioavailability

Stage	Knowledge gap	Examples	Reference examples
Food matrix	Physical state/compartmentalization of phytochemicals	Crystallinity of carotenoids in chromoplasts versus chloroplasts, polyphenols bound to cell wall (NEPP)	31, 107
Food preparation	Effect of cutting, mashing, grinding, peeling, trimming	Enzyme activation (e.g. polyphenol oxidase, alliinase) Concentration differences in morphological parts	42, 43
Food processing	Effect of cooking (heat, temperature, time, blanching)	Enhanced carotenoid content with mild conditions but decreased with severe treatments	16, 52, 53
	Refining processes	Bleaching, deodorization: decreasing carotenoid content Milling enhancing polyphenol extractability through surface area	40, 41, 42
	Nonthermal processing (HPP and PEF)	Both positive (polyphenols) and contradictory effects (carotenoids)	67, 69
Food mixtures	Effect of mixed or real meals	Enhanced availability of polyphenols with presence of sugars, ascorbic acid, fat. Stabilizing effects of polyphenols on other phytochemicals?	77, 81, 193
Gastric digestion	Effect of pH, depolymerization of large polyphenols, binding effects	Hydrophobic interactions, hydrogen bonding of polyphenols to proteins	19
Small intestine	Micelle formation Role of uptake transporters	Transition from matrix to oil phase, micelle size, number, and stability St. John's Wort components increased P‐gp function	22, 83
	Efflux transporters cell→gut lumen	Efflux transporters such as P‐gp, BCRP blocked by several polyphenols, e.g. flavonoids	124
	Efflux transporters cell→basolateral	Effect of polyphenols on efflux transporters such as MRP3	194, 195, 196
	Transporters gut lumen→cell	SRB‐1, CD36, NPC1L1, ABCG5/G8: affecting carotenoid uptake – influence of polyphenols	19, 197, 198
Colon	Phase I/II interactions	Piperine increased curcumin absorption	146
	Influence of microflora Colonic absorption	Metabolite formation, absorption of cleavage products?	102, 172, 190
	Metabolites and phase I/II products	Metabolite information limited by lack of standards	160, 161
Tissues	Biotransformation, phase I and phase II metabolism	Need to develop/increased availability of more physiological cell models (liver stem cells, co‐culture cell models) to study metabolites	125, 152, 153, 154, 155
	Interaction with transporters in certain tissues (blood–brain barrier, placenta, testis), or excretory organs (liver, kidney)	Role of MRPs, MCTs, CD36	140, 184, 185

ABCG5/G8 = ATP‐binding cassette sub‐family G, member 5/8; BCRP = Breast cancer resistance protein; CD36 = cluster of differentiation; HPP = high pressure processing; MCTs = monocarboxylic acid transporters; MRP = multidrug resistance protein; NEPP = non‐extractable polyphenols; NPC1L1 = Niemann‐Pick C1‐Like 1; PEF = pulsed electric fields; P‐gp = P‐glycoprotein; SRB‐1 = scavenger receptor class B member 1.

**Figure 1:**
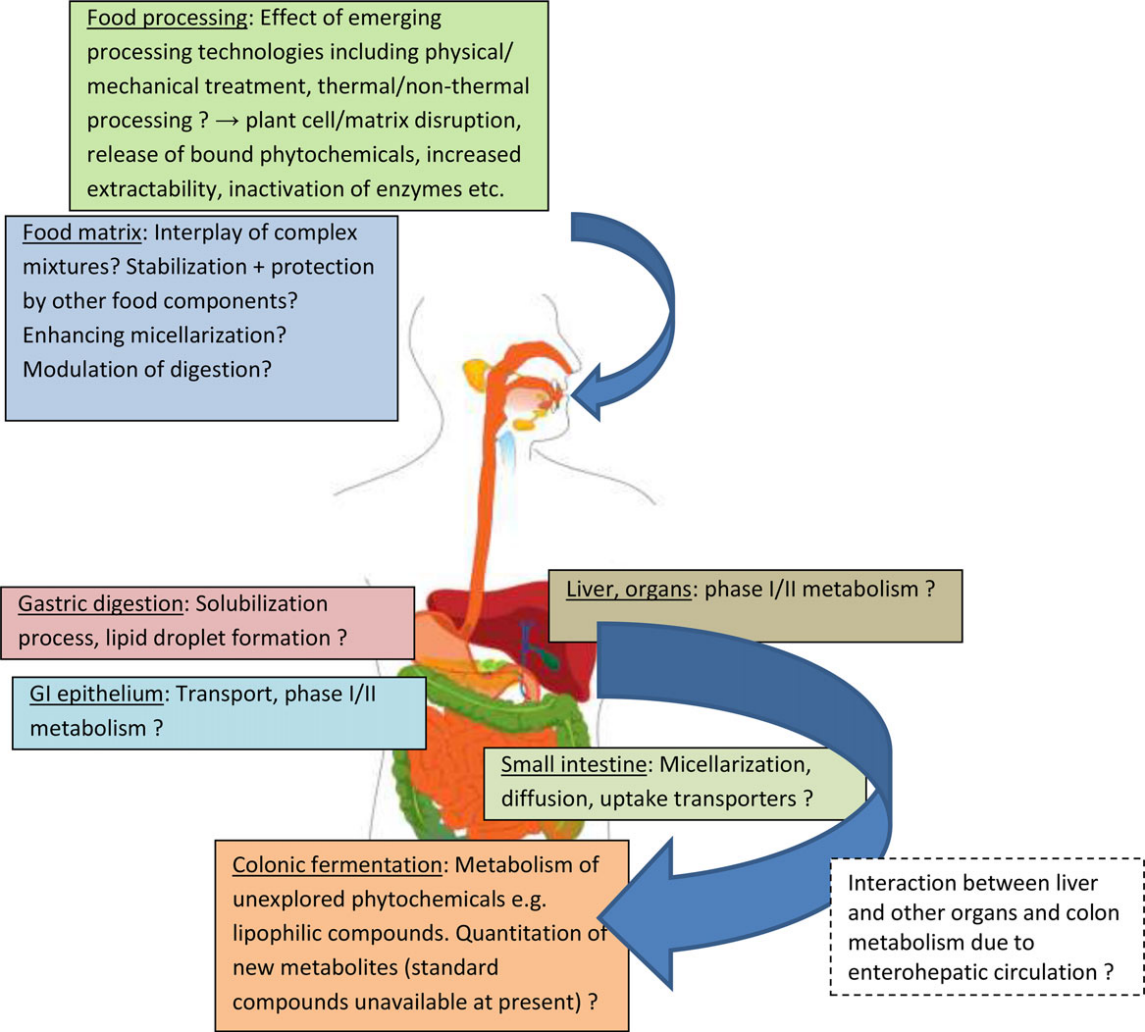
Overview of important stages during the digestion of food items and open questions related to phytochemical bioavailability.

In this paper, we aim to highlight gaps that have received either limited attention or which are far from being understood, but which may play pivotal roles in the bioavailability of phytochemicals (Fig. 1). To allow for better focus, this review will concentrate on polyphenols and their metabolites as the most abundant water‐soluble compounds, and carotenoids as the most abundant lipophilic secondary plant compounds. For a more comprehensive overview on bioavailability aspects of specific compound classes, the reader is referred to other articles 22, 27, 28.

## 2 Food processing and matrix effects

Bioaccessibility, and further bioavailability, of phytochemicals starts with their content and composition of the raw plant material and how it is processed. The food matrix and structure and type of processing can have both positive and negative effects 29. Plant matrix disruption and cell cluster disintegration due to applied processing steps are the main prerequisites for phytochemical liberation and bioaccessibility, but they may also lead to oxidation and/or degradation, thus potentially counterbalancing each other.

To understand the release of bioactive compounds during digestion, it is also important to know where they are located in the tissue. Carotenoids are found either in the chloroplast membrane or in chromoplasts 30. The different physical forms of carotenoids in plant chromoplasts (crystals in tomato, lipid‐dissolved in papaya and liquid‐crystalline in mango) have major impacts on their liberation efficiency from the food matrix 31. Polyphenols are generally present in vacuoles and the apoplast of plant cells, in conjugated form with mono‐ and polysaccharides, and proteins. Although studies have focused mainly on the cellular localization of carotenoids, polyphenols, and more rarely on glucosinolates, more research on their localization in different foods is needed. Little is known about other phytochemicals.

### 2.1 Effects of physical and mechanical treatments

Food preparation (such as grating, chopping, cutting, slicing, trimming, mashing, and juicing) may have great influence on the bioaccessibility of nutrients and bioactive compounds 30, 32. The recovery of phytochemicals from intact, minimally processed fruits and vegetables can be different from juices as components must be released from the tissue matrix before being subject to degradation reactions, whereas those in the juices are subject to degradation from the offset. However, carotenoid bioacessibility was greater from juiced than from raw or cooked puréed tissue, as chopping and homogenization disrupted the plant matrix 32. The levels of carotenoids in sea buckthorn berries were lower if the berries were extracted in water or juiced than after direct acetone extraction. This appears to be due to the complexation of carotenoids with pectins 33, and similar interactions may also occur between pectins and polyphenols, such as anthocyanins, during digestion 34.

Other phytochemicals are subject to endogenous enzymatic digestion. Chopping or crushing raw garlic releases alliinase which produces allicin, which breaks down to diallyl sulfides and then other metabolites, which may influence chronic diseases and certain cancers 35, 36. Similarly, the glucosinolate glucobrassicin, which is found in cruciferous vegetables, is hydrolyzed when plant cells are damaged 37. On the other hand, flavonoids seem relatively stable to mechanical processing 38. Peeling and trimming steps may also influence phytochemical content. Trimming green leafy vegetables is known to be a main factor influencing carotenoid concentrations, since they are nearly exclusively present in leaves 39. Also industrial refining processes, e.g. as applied to vegetable oils and cereals, may lead to high losses in phytochemicals. Refining of palm oil decreased carotenoids by 99%, and polyphenols by 23% 40, 41.

Milling of cereals and grains has a great influence on phenolic acids 42. It is well known that milling of the whole grain results in higher amounts of phenolics than conventional milling resulting in white flour 43. Many studies have investigated the effects of refining on phytochemicals in edible oils and cereals but similar studies on vegetables and fruits (except juices) are largely absent.

In conclusion, although there are many studies reporting effects of food preparation on the bioaccessibility of polyphenols and carotenoids, understanding the mechanisms of chemical or enzymatic actions still requires extensive work. In addition, as novel methods are developed, further studies on how these processes impact bioaccessibility and bioavailability are warranted.

### 2.2 Effects of thermal and nonthermal treatments

Studies of thermal treatment on phytochemicals in foods have mainly focused on polyphenols, carotenoids and glucosinolates. Many reports indicate that thermal processing increases levels of free polyphenols 44, 45, 46, 47, possibly due to the release of bound phenolics due to the breakdown of cell constituents, perhaps through releasing nonextractable polyphenols (NEPP). However, also polymerization/oxidation reactions may be responsible for apparent increases 44, 45, and the nature of these released polyphenols requires investigation. Similarly, carotenoid availability often increases after heat treatment 48, 49, 50, 51, probably due to enhanced extractability following maceration of cells. However, severe heat treatment such as baking/sterilization of tomatoes 52, 53 or boiling of chili peppers 54 caused significant losses. Losses of glucosinolates in *Brassica* vegetables depended on processing time, type of vegetable, degree of cellular damage, and inactivation of myrosinase 55, 56, 57, 58, 59, 60. Overall, further research is required to better explain how thermal treatments impact bioaccessibility and bioavailability.

Nonthermal processing technologies have been revealed as useful tools to extend shelf‐life and to preserve nutritional and functional characteristics of fruit and vegetable products 61. However, there are scarce data on the effect of these emerging technologies on bioaccessibility and bioavailability of bioactive compounds. Considering bioaccessibility, studies have mainly focused on the effect of high pressure processing on carotenoids, with contradictory results. Some show improvements 16, 62, 63, 64, 65, 66, possibly related to the disintegration of cell clusters and disruption of cells containing carotenoids. Beneficial effects of a subsequent thermal treatment may weaken the physical barriers that enclose carotenoids and accelerate pectin degradation by β‐elimination, resulting in cell wall softening. Decreased carotenoid bioaccessibility has been noted in some vegetables and fruits and may be due to firmer texture and/or trapping of carotenoids within fiber networks 62, 63. Thus, processing variables (e.g. particle size, temperature, pressure applied) need to be carefully controlled, and there is a need for harmonization and validation (by comparison with in vivo assays) of the in vitro digestion models used.

Regarding human bioavailability, high pressure processing increased absorption of orange juice flavanones over conventionally pasteurized orange juice 67. No data are available for carotenoids. To our knowledge, the effect of pulsed electric fields has been investigated only on vitamin C bioavailability in humans from orange juice 68 and Mediterranean vegetable soup 69. No studies addressing the effect of pulsed electric field on carotenoid and polyphenol bioaccessibility and bioavailability are available. Other nonthermal processing technologies such as ultrasound 70, food irradiation, light pulses or oscillating magnetic fields, have received little or no attention in terms of their effect on bioaccessibility and bioavailability of phytochemicals. Thus, more studies in this field may be required especially as more novel processing techniques are being developed.

## 3 Mixtures of authentic food matrices

There has been comparatively little effort into assessing the effects of characteristic combinations of foodstuffs or realistic meals on the bioaccessability of phytochemicals. However, studies have shown that polyphenols from foods in combinations can have very different bioaccessibilities. Studies with raspberry juice showed that the addition of ice‐cream markedly reduced the recovery of total anthocyanins 71, whereas a wheat‐based breakfast cereal did not influence recovery. Co‐digestion with blueberries ± oat meal and/or milk revealed that milk reduced the recovery of total anthocyanins and total phenols 72 and co‐digestion of nuts and dried fruits yielded lower levels of total available phenols after simulated digestion than nuts or fruits alone.

The addition of bovine, soy and rice milks, ascorbic acid or citrus juices increased the bioaccessibility of galloylated green tea catechins (EGC, EGCG, EC, and ECG), by stabilization and protection from degradation at alkaline pH 73. The simultaneous presence of sucrose and ascorbic acid in green tea increased EGC and EGCG bioaccessibility, uptake in Caco‐2 cells and bioavailability in rats 74. Although the addition of skimmed milk reduced recovery of green tea catechins after in vitro digestion, the uptake of catechins by Caco‐2 cells was increased 75. This suggests that catechins bound to the milk proteins after in vitro digestion were available for uptake by the Caco‐2 cells. This may explain the lack of difference in serum/plasma bioavailability of tea catechins in studies where subjects were given tea with or without milk 76. Similarly, chocolate containing higher sucrose levels increased plasma concentrations of metabolites derived from catechins compared to dark and milk chocolate in rats 77. In humans, sucrose but also solid/beverage format influenced various aspects of the bioavailability of flavan‐3‐ols from commercial cocoa based products 78. Dietary fats can increase polyphenol bioavailability in humans by increasing absorption, possibly by enhancing micellarization in the small intestine 79, 80, as noted for carotenoids. For example, higher fat content increased the stability of cocoa procyanidins during in vitro digestion 81. This is particularly relevant as screening of different formulations (or different plant varieties) for the bioaccessibility of specific phytochemical components has become more common and it is possible that variations in other macronutrients influence the outcome. Using experience gained in pharmaceutical applications, strategies may also be designed to improve polyphenol bioavailability by co‐administering phenolics with compounds which modulate gut and/or liver metabolizing enzymes 80, 82, 83.

There is definitely more scope for the study of food mixtures that are more realistic of real meal choices. Complex models developed by the pharmaceutical industry to assess the digestion behavior of different drug formulations 84, 85 could be applied to estimate digestion of foods. Despite the increasing complexity achieved by in vitro models, they remain simple compared to in vivo models. Typical limitations of in vitro models include: absence of host response factors, poor stimulation of complex mechanical forces and gastric emptying, absence of microbial flora, low level of integration into an overall digestive process, general adherence to healthy/average conditions, and limited correlation to in vivo situations 86. Nevertheless, some examples have indicated a reasonable correlation between bioaccessibility studies and the situation in vivo. For lipophilic compounds such as α‐ and γ‐tocopherol, β‐carotene and lycopene 87, β‐cryptoxanthin 88 or phytosterols 89, high correlations have been found, indicating that estimating in vitro bioaccessibility (solubility/micellarization) can be indicative of the amount available for uptake in the GI tract in vivo. For polyphenols, comparisons to in vitro data have been allowed by studying ileostomists 15. In addition, combining in vitro digestion models and human intestinal cells (e.g. Caco‐2 cells with or without a layer of mucus‐producing cells such as HT‐29 MTX), carotenoid uptake 90, 91, 92 qualitatively and quantitatively correlated well with human data. Investigators using in vitro methods must consider how to adapt the digestion conditions according to the composition of the sample and/or to food components, seeking a balance between technical simplification and accuracy, considering the in vivo situation as reference.

However, the release of phytochemicals from different foods is complex and involves large numbers of variables 93, 94, 95. Furthermore, the transfer of phytochemicals from authentic food matrices will be even more complex due to a greater number of potentially rate‐determining processes. Digestion and absorption rates are limited by physical processes, operating within and between the different phases of the liquid and solid phases of the digesta 96. Using models of the human stomach 95, 97, distinct disintegration profiles and kinetics have been found for different food categories (e.g. meat products, nuts, fruits, baked and fried products, etc.). The digestion process in terms of secretion of gastric fluids and enzymatic degradation of macronutrients is well understood, but further detailed investigations are needed to understand lipid absorption and to predict responses in realistic meals. Further information on the release of bioactive compounds should provide a better understanding of how to combine foods and design meals to improve bioavailability and enhance potential bioactive effects.

Encapsulation may enhance the bio‐effectiveness of phytochemicals, and the choice of encapsulation agent can influence stability/recovery after digestion 98. For example, Haratifar et al. 99 found that casein micelles were effective encapsulation agents for EGCG. A novel approach involving biosorption of phenolics to *Saccharomyces cerevisiae* significantly improved bioaccessibility of total phenolics, presumably as the yeast provided a protective carrier against degradation at neutral pH 100.

The possibility that certain phytochemical classes could protect other phytochemicals is an interesting, but largely under‐researched area. The fact that the stability of individual phytochemical classes may be dependent on the overall phenolic composition of the sample has been raised in studies of berry polyphenol bioaccessibility 101, 102. This interdependence could be examined in studies where blends of juices are tested for bioaccessibility of a range of phytochemical components such as vitamin C, carotenoids, and polyphenols 103. The possibility that polyphenols may sacrificially protect carotenoids or vitamin C (or vice versa) could also be examined. For example, the effect of anthocyanins on carotenoid stability could be examined in comparative studies of purple tomatoes and related red tomatoes 104 or indeed in the recently genetically modified anthocyanin‐accumulating tomatoes over their non‐genetically modified counterparts 105. Combining foodstuffs, therefore, may provide another route to enhance the bioaccessibility of specific health beneficial components. Vice versa, it has been claimed that polyphenols may modulate the digestion of macronutrients (such as starches or fats) through inhibition of their digestive enzymes 106, which could possibly modulate their own stability in the gut. The possibility that the underestimated NEPP content of fruits/vegetables 107 could influence the activity of digestive enzymes or limit the access of certain phytonutrients to digestive processes could be examined in carefully designed studies where the effect of in vitro digestion on the phytochemical profile of whole fruit/vegetable purees was compared to extracts and cross‐compared to re‐combined samples containing NEPP plus extracts.

There has been a general shift away from measuring the levels of total phytochemical classes (e.g. total carotenoids and phenolics) to targeted analysis of compositional changes in specific components during digestion, as this is more relevant to the potential health benefits attributable to specific phytochemicals. However, greater value could be obtained by using an untargeted, metabolic profiling approach which could also identify potential breakdown products and other novel components.

## 4 Gastric phase and small intestine

The absorption of secondary plant components includes several phases (outlined below), and prominent gaps in our knowledge include factors influencing solubilization, micelle formation (of apolar compounds), diffusion to the unstirred water layer (including the influence of mucus), transporters involved in the uptake of phytochemicals, and factors affecting phase I/II metabolism and efflux pumps.

### 4.1 Release from the food matrix

Aspects impeding the release of phytochemicals include large particle size 108, high meal viscosity 18 reducing the transfer of lipophilic compounds to micelles and hindering interactions between lipase and oil droplets 109; or the presence of physically inaccessible forms such as NEPP 19, 110. As the majority of micronutrients and phytochemicals are presumably taken up in the small intestine (in their native form), and the epithelial “leakiness” decreases toward the colon 111, it is important that compounds are bioaccessible at this stage. Many polyphenols may not be detectable in the native matrix following chemical extraction, but may be released during digestion in the small intestine, such as those bound covalently or occluded by e.g. in accessible starch 112, though colonic fermentation may further result in the breakdown of NEPPs 113. Standardized methodologies which recognize and examine the potential contribution of NEPPs are required. It also has to be noted that polyphenols have the ability to reduce the activity of digestion enzymes (e.g. pepsin, lipase), thus high concentrations of polyphenols may reduce liberation of lipids and proteins, increasing the nondigestible bulk and in turn may result in increased amounts of polyphenols passed onto the colon 19.

### 4.2 Solubilization and micellarization

Solubility is not an issue for most polyphenols, but more lipophilic compounds such as carotenoids 114, triterpenes 115, and phytosterols 116 require emulsification/micellarization before uptake. It remains largely unknown to what extent digestion and food matrix influence micelle formation or size. Large micelles would compromise diffusion and subsequent release of apolar compounds. Micelle diameters of approximately 6–8 nm 117, 118 have been measured, but the relation of micelle size, shape, or constituents, and cellular uptake has never been studied in detail. Also, surprisingly little is known about the influence of dietary lipids and their digestion on polyphenol uptake 19, though there are indications that certain apolar polyphenols (e.g. curcumin, resveratrol, quercetin aglycones) are incorporated into micelles, and that lipid‐rich foods may enhance their bioavailability 79, 119, 120, 121.

A poorly understood factor in bioavailability is the role of brush border membrane enzymes, i.e. maltase, lactase‐phlorizin‐hydrolase, sucrase‐isomaltase, and peptidases 122. Lactase‐phlorizin‐hydrolase may play a crucial role in cleaving polyphenol glycosides, resulting in the uptake of free aglyones 123, 124. However, it is not certain whether cleavage occurs at this stage, by cytosolic‐beta‐glucosidase 19, or by colonic microbial action. The presence of esterases in the brush‐border, surely present in the enterocyte 125, has been speculated on, which would influence polyphenol esters such as chlorogenic acid, or xanthophyll esters, though cholesterol esterase may also act on the latter 126.

While proteins can exert negative effects on polyphenol bioavailability 127, the interaction of apolar phytochemcials and proteins during digestion has never been studied systematically, though certain proteins may aid in the emulsification of lipid soluble phytochemicals 128. Contrarily, a positive effect of sugars on polyphenol glucoside uptake [via stimulation of sodium‐glucose linked transporter 1 (SGLT‐1)] has been suggested 129. The effect of dietary fibers is assumed to be negative, due to gel formation, enhanced viscosity, or binding and entrapping of phytochemicals, but the effects of soluble versus insoluble, or prebiotic fiber are largely unknown. Other interactions with the food matrix exist during digestion, such as high concentrations of minerals impeding micelle formation 130, but their relevance in bioavailability remains unclear.

### 4.3 Cellular uptake

Prior to reaching the cellular surface, diffusion through the mucus 122 is required, though properties influencing diffusion are poorly understood. Viscosity and particle/micelle size of the digesta are expected to play a role. Porcine trials have shown that smaller particles diffuse more readily through the mucus layer 131; and it may be assumed that apolar compounds are likewise hindered 132. More sophisticated model studies including mucus‐producing cells, such as HT‐29‐MTX, are still rare but warranted.

Cellular uptake can occur by transcellular or paracellular routes. The latter is reserved for rather polar and small molecules <600 Da 133, i.e. ions, water, sugars, etc., as these pass through the tight junctions of the epithelium. Most phytochemicals are taken up via transcellular transport. Certain compounds (such as apple polyphenols) increase tight junction functionality 134, possibly via altered cellular signaling transduction pathways 135. On the other hand, certain fatty acids (such as caprylic acid) and high molecular weight polyphenols reduced tight junction barrier function, and may enhance uptake of other small compounds 134, 136, 137.

Transcellular uptake can occur by facilitated, active means or via passive diffusion. The latter is reserved for small and apolar molecules, as these readily pass through the cell membrane 138. Passive diffusion has been suggested for carotenoids 22, 23 and some apolar polyphenol aglycones 19. At low concentrations, presumably similar to levels of an average mixed diet, these compounds may also be taken up by carriers, actively or by facilitated diffusion. Over 400 transmembrane carrier proteins are known 139. Many polyphenols can inhibit these transporters, but stimulation was also described 140 and effects may depend on the polyphenol type, its concentration, exposure time, cell type, and substrate. For carotenoids, high concentrations of beta‐carotene reduced lutein uptake 141, which may be explained by higher affinity to transporters such as cluster of differentiation 36 (CD36) or unknown types 142. Enhanced uptake of polyphenol glucosides by sugars via SGLT‐1 activation 19, 143 is known, but the involvement of gut transporters other than SGLT‐1 and mono‐carboxyl‐transporter 1 (MCT1) for polyphenols is not certain, and most polyphenol aglycones are assumed to be taken up by passive diffusion 124.

### 4.4 First pass metabolism

A large number of metabolites can be formed following phase I (e.g. reduction/oxidation, methylation, hydroxylation, hydrolysis, e.g. via cytochrome P450‐dependent mixed‐function oxidases (CYPs) and catechol‐O‐methyl‐transferase) and phase II metabolism (e.g. glucuronidation by uridine‐5’‐diphosphate glucuronosyltransferase, and sulfation via sulfotransferases), in human enterocytes 144, 145. Certain phytochemicals can up‐ or downregulate these enzymes, influencing the availability of the native compounds. For example, uptake of curcumin was approximately 20‐fold enhanced when co‐administered with piperine in humans 146. High doses of certain polyphenols may override phase I/II metabolism, though not much is known on thresholds in this respect 19. Such interactions are certainly a gap in our knowledge. For carotenoids, cleavage by beta‐cartotene oxygenase 1/beta‐carotene dioxygenase 2 results in symmetric/asymmetric cleavage, producing apo‐carotenals. However, these reactions are far from quantitative, and appear to depend on genetic factors and the type of carotenoid 23. The bioactivities of apo‐carotenals (except retinols) are poorly comprehended, though they may be highly effective, e.g. in activating nuclear receptors 147. Detecting small amounts of these compounds is difficult, and suitable standards are often not commercially available.

### 4.5 Transport through the epithelium

Many phytochemicals are treated as xenobiotics, expelled from the cell, typically by increasing their polarity and via efflux transporters. These transmembrane proteins are typically ATP‐dependent efflux pumps. Little is known on the specificity of these transporters, and less on the potential of phytochemicals or nutrients to block them, which could result in enhanced bioavailability. It is assumed that P‐glycoprotein, MRP2 (multidrug resistance protein 2), and breast cancer resistance protein (BCRP) are the most important transporters, and that these may be competitively inhibited by polyphenols, perhaps by blocking ATPase 19. The transport from the cell to the basolateral side (i.e. bloodstream or lymph) is even more poorly understood. Compounds that could increase or decrease MRP3 (or MRP1, MRP4, and possibly several MCTs), could affect polyphenol uptake, as e.g. demonstrated by mice overexpressing MRP3 and showing high resveratrol bioavailability 148. It is assumed that carotenoids can be transported back into the lumen by scavenger receptor class B member 1 and possibly ATP‐binding cassette subfamily G, member 5/8, but more research is needed. Transport through the cells, e.g. by transporters such as fatty acid binding proteins is assumed, but not confirmed 23. It can be further assumed that proteins involved in chylomicron generation [i.e. microsomal triglyceride transfer protein, apoB48, apoAIV, and Sar1b 23, 149, also influence carotenoid transport. For polyphenols, transport through the cells is not understood, and may occur primarily by diffusion.

## 5 Metabolism in the colon and other organs

### 5.1 Metabolism of polyphenols

In general, polyphenol metabolism is fairly well known due to their xenobiotic nature. Polyphenols undergo metabolism in intestinal and liver tissues and by colon microbiota 15. In fact, it seems that the colon may be the major important site for polyphenol uptake, at least for orange juice rich in hesperitin and naringin 150, and studies with ileostomists suggested significant absorption from the colon 151. Phenolic compounds are glucuronidated and sulfated in the liver and intestinal tissues, and these metabolites are found in body fluids 125, 152, 153, 154, 155. Hepatic metabolites can be recycled back to the small intestine through biliary excretion 27, 156, 157, 158, 159 and end up in the colon, where they are deglucuronidated by microbial α, D‐glucuronidases before ring fission 160, 161.

Sophisticated methods for studying enterohepatic circulation, including humans, using a perfusion technique, have been published 162, 163, in addition to various articles on tissue metabolites of phenolic compounds 164, e.g. flavanol monomers and tea polyphenols. However, the enterohepatic circulation of colonic metabolites requires further investigation and hepatic metabolism of colonic metabolites of plant phenolic compounds should be addressed in the future 162. Furthermore, Monagas et al. 165 suggested that microbial metabolites may act as signal molecules, and their action should be taken into account in more detail in future investigations.

Absorbed microbial‐derived metabolites (such as enterolignans and dihydroxylated compounds such as methyl catechol) can be subjected to further glucuronidation, methylation, sulfation, or glycination in the liver, while phase I metabolism (oxidation/reduction reactions) seems to occur to a lesser extent 27. The interplay between the liver and the colon, the enterohepatic circulation, leads to a long residence time (up to 24–48 h) in the blood 157, 162, 166, 167, 168, which can go unobserved unless longer sampling of blood or urine is carried out 164, 169. Finally, the phenolic microbial metabolites are distributed to tissues and are excreted via urine, partly as free but mainly as hepatic conjugates, depending on the structure of the parent backbone 125, 157, 158.

One of the major gaps is the lack of knowledge concerning hepatic conversion of small colon‐derived phenolic acids, which may be missed in analyses of human body fluids or cell lines due to their hydrophilic nature. Extraction of samples may also cause bias, as Sawai et al. 157 elucidated the ratio of excreted conjugated and free phenolic acid colon‐derived metabolites from quercetin derivatives in urine, by using water saturated ethyl acetate to enhance the yield of polar conjugates.

Although human studies are more relevant, mechanisms of action can be studied using in vitro human‐based cell model systems designed to study properties of drugs 27. In recent years, novel cell lines and culture strategies have helped in overcoming the scarcity of human liver material and problems in maintaining the expression and function of metabolizing enzymes 170. The advent of human stem cell derived hepatocytes will potentially provide an unlimited source of human hepatocytes 171. Human hepatocyte three‐dimensional models, with complete hepatic metabolizing enzymes, transporters, and cofactors, may be applicable to metabolite profiling, pathway identification, CYP450 inhibition, CYP450 induction, and uptake and efflux transporter inhibition by polyphenols and their metabolites.

The lack of standards for metabolites causes limitations to study both polyphenol and phytosterol metabolism. Chemical procedures and biochemical labeling tools are available for the synthesis of many conjugated metabolites 172. However, the absence of standards limits the use of some analytical methodologies, and frequently enzyme treatment is used to overcome the problem of hepatic conjugative metabolism. For example, conjugated flavan‐3‐ol metabolites are rarely available commercially, and most studies have analyzed plasma and urine samples after treatment with glucuronidase/sulfatase, providing data only for the aglycones 164. However, some sulfate conjugates are resistant to enzyme hydrolysis, thus, this methodology may underestimate bioavailability 173, 174. This is the situation for estimating epicatechin bioavailability from cocoa products. Direct analysis of the individual epicatechin metabolites by LC–MS/MS may overcome this problem, however, the chirality of the aglycone will still be unknown, and it is plausible that the enantiomers could differ in their biological activity. Each flavan‐3‐ol and each associated phase II conjugate can, in theory, occur as four enantiomers, with the (+) and (–) forms resolvable only by chiral chromatography, and so far as we are aware there are no reports of chiral analysis of conjugated metabolites in urine or plasma 164.

However, targeted LC‐MS analytical approaches can be used for identifying many metabolites by exact mass, if a theoretical prediction of possible metabolites and conjugates based on expected metabolism is available 154. Nontargeted metabolomic profiling can find metabolites based on structural similarity to the parent compound, if the mass spectra can be coupled with a compound library. Profiling of phenolic metabolomes has been assessed by NMR, GC, LC and 2D‐GC with TOF mass detection coupled with a compound library 25, 175, 176, 177, 178.

### 5.2 Metabolism of lipophilic compounds

Regarding lipophilic bioactive compounds, serum phytosterol bioavailability is reported to be below 10%, suggesting that they may reach the colon and subjected to microbial metabolism 179. The pathway of microbial transformation from sterol to stanol form in humans was reported in the 1970's, but the kinetics of the reaction steps and metabolites in the large intestine have not been studied systematically 180. For carotenoids, the liver stores and distributes carotenoids to other tissues, but the mechanisms are not known accurately 181. However, intervention studies have shown that increased dietary intake of carotenoids influences serum concentrations more than colon concentrations 182, suggesting that absorption occurs mostly in the small intestine. In addition, metabolites of lycopene produced in vivo also occur naturally at low concentrations in tomato, which causes difficulties in the differentiation of the origin of the metabolites 183.

### 5.3 Transport, tissue distribution, and mechanism of action

Transport of metabolites and their parent compounds to tissues has not been studied adequately. This is important, since it is the key action to ensure the desired biological activity. There is a lack of information whether conjugated polyphenols can prolong their residence time in the body by modulating the activity of the transporters in the excretory organs. A recent study of transmembrane transport of flavonoids and some of their methylated and glucuronidated metabolites using human cerebral microvessel endothelial cells as a blood–brain barrier cell model 184 showed that the metabolites were transported in a time‐dependent manner and showed higher transport efficiency than the native flavonoids and were not further transformed by the cells. Polyphenols affect their own metabolism and tissue distribution, due to their capacity to modulate the activity of xenobiotic metabolizing enzymes and transporters, modulating their own concentration‐time profiles in the body and also promoting alterations in drug or toxin pharmacokinetics, the mechanisms of which are not known 140, 185. Therefore, additive, synergistic, or antagonistic effects of xenobiotics (dietary phytochemicals, drugs, and toxins) need to be addressed. For example, the downregulation of cytochrome P450 3A4 by grapefruit juice and its polyphenols, enhancing the concentration of several pharmaceuticals and increasing their effects is well known 186. Furthermore, the knowledge of pharmacodynamics and tissue distribution for most polyphenols and their metabolites is still lacking. It would also be relevant to study individual variation in the tissue distribution and the potential bioactivity caused by genetic variation in pathways related to bioavailability and transport 187.

In vitro models for preclinical research using stem cells, and patient‐specific induced pluripotent stem cells and reprogrammed somatic cells from patients are already applied in disease modeling and drug discovery, and may be applicable to test polyphenol metabolite health benefits. Microengineered physiological systems, also known as “organs‐on‐chips”, can reconstitute physiologically critical features of human tissues and their interactions 188. The nematode *Caernohabditis elegans*, with the presence of tissue and organ systems, is increasingly used as an in vivo model and has been employed to study the metabolism of methylated catechin derivatives and their biological effects on oxidative and thermal stress resistance 189. Thus, novel cell assays and nematode in vivo assays may be applied to study mechanisms of action of the parent compounds versus their liver/colon metabolites, although it should be noted that in vivo models such as *C. elegans* or *Drosophila* (like mice) may produce quite different metabolites from ingested phytochemicals than humans.

Only a few studies have combined the assay of biological effects of bioactive compounds or their colonic metabolites from whole foods or their extracts with an in vitro GI digestion process with/without colonic fermentation 102, 172, 190, or compared the biological activity of metabolites to their parent compound 178. Furthermore, the synergistic effects of the phytochemical metabolite pool, including the interplay between liver and colon microbiota should be studied. Finally, cell‐based assays measuring the bioactivity of the metabolites at relevant concentration are scarce 102, 191, 192. In the future, bioactivity assays to mimic the actual in vivo situation in the corresponding tissue, should use metabolite pools from whole foods, at tissue‐tolerant concentrations.

## 6 Conclusion

In this position paper, we have tried to highlight gaps of knowledge with respect to selected phytochemicals. It has to be noted that the data presented represent a somewhat simple and general paradigm for phytochemicals, and that, due to the very large variety and properties of secondary plant compounds, the statements cannot be generalized toward all groups of phytochemicals. In addition, some of the missing aspects discussed in this paper may currently be tackled, but have not been published.

However, while recent work has greatly enhanced our insight into the metabolism and bioavailability of a range of phytochemicals, many factors governing matrix release, solubilization, cellular uptake, and biotransformation remain poorly understood. Major aspects which deserve more attention when estimating bioavailability aspects include effects of innovative processing techniques, synergistic effects of mixed/whole diets, factors effecting micelle formation, co‐constituents influencing influx and efflux via transporter systems or altering phase I/II metabolism, as these have often been overlooked or excluded from consideration, in part due to difficulties to include their study in vivo or in vitro. In the future, enhanced availability of analytical possibilities to investigate these aspects such as through broader availability of instruments to measure food texture, visualization of micelles (TEM, Mastersizer), improved cell models of absorption and metabolism (mucus producing, liver cells, 3D models), ways to produce knock‐out variants (e.g. of certain transporters) in animal models (nematodes, mice, etc.) to study pathways of absorption and bioactivity of metabolites, and improved chromatographic techniques and commercial availability of metabolites (e.g. sulfates and glucuronides) will aid toward an improved understanding of these important aspects of bioavailability.

*The authors have declared no conflict of interest*.
